# Supplementary material for: HIV-1 promotes ubiquitination of the amyloidogenic C-terminal fragment of APP to support viral replication
Source: Nat Commun. 2023 Jul 15;14:4227. doi: 10.1038/s41467-023-40000-x (PMC10349857; doi:10.1038/s41467-023-40000-x)
Supplement: Supplementary file 1 — Supplementary Information [file 41467_2023_40000_MOESM1_ESM.pdf]

Supplementary Fig. 1

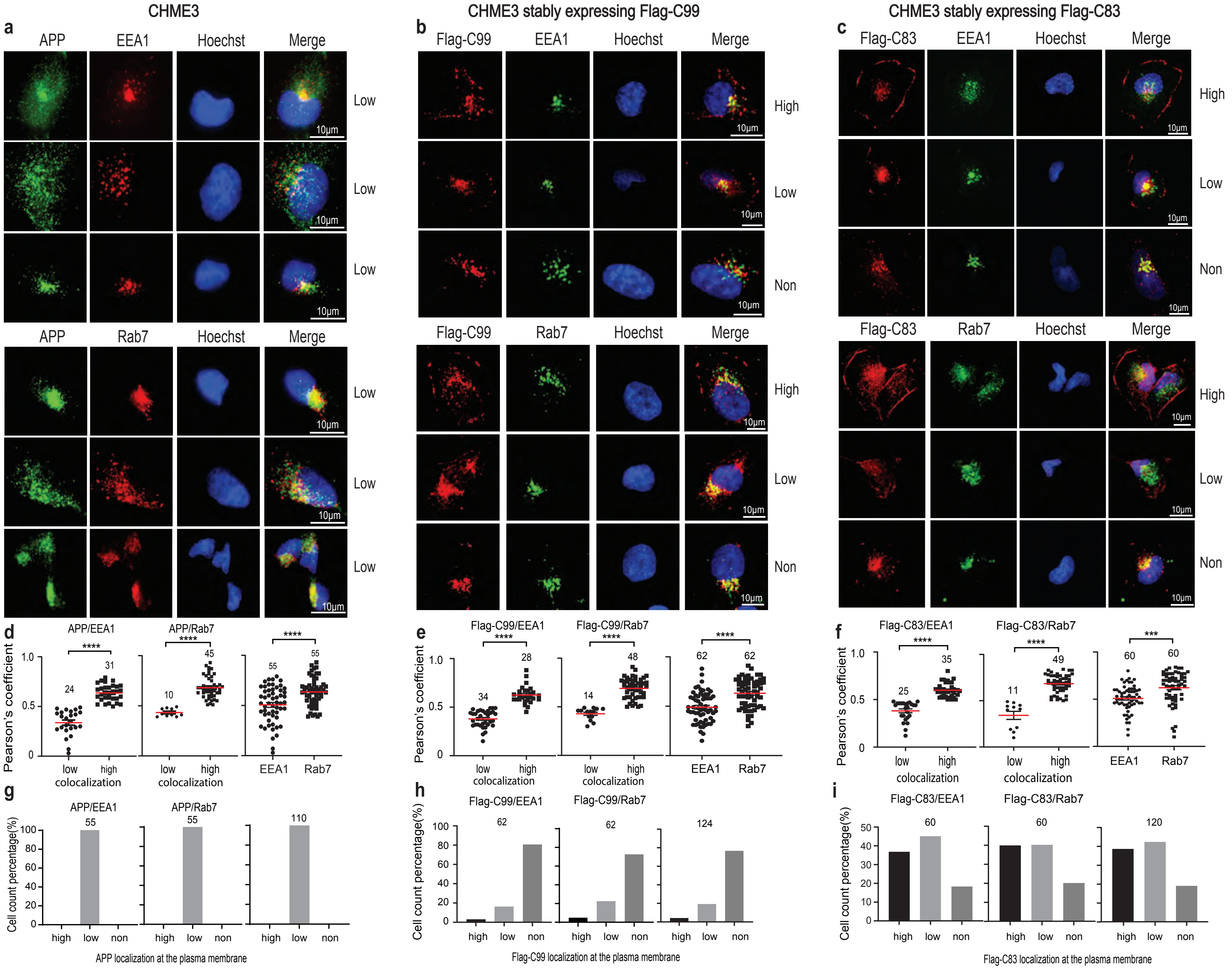

Supplementary Fig. 1. The distribution of endogenous APP and exogenous CTFs in CHME3 cells. a-c. Representative images of endogenous APP (**a**), Flag-C99 (**b**) or Flag-C83 (**c**) along with EEA1 (upper panels) or Rab7 (lower panels) staining in unmodified CHME3 (**a**) or CHME3 cells stably expressing Flag-C99 (**b**) or Flag-C83 (**c**). Nuclei was detected using Hoechst. **d-f.** Quantification of the colocalization of APP (**d**), Flag-C99 (**e**) or Flag-C83 (**f**) and EEA1 or Rab7 using Pearson's Correlation Coefficient; mean with SEM using unpaired two-tailed *t* test with Welch's correction (APP/EEA1 and APP/Rab7 in d, Flag-C99/Rab7 in e) or unpaired two-tailed *t* test for the remaining graphs, ***p < 0.001, ****p < 0.0001. Number of cells analyzed is indicated. **g-i.** Percentage of cells with APP (**g**), Flag-C99 (**h**) or Flag-C83 (**i**) localization at the plasma membrane for each group in **d-f**, respectively. Number of cells analyzed is indicated. Source data are provided as a Source Data file.

Supplementary Fig. 2

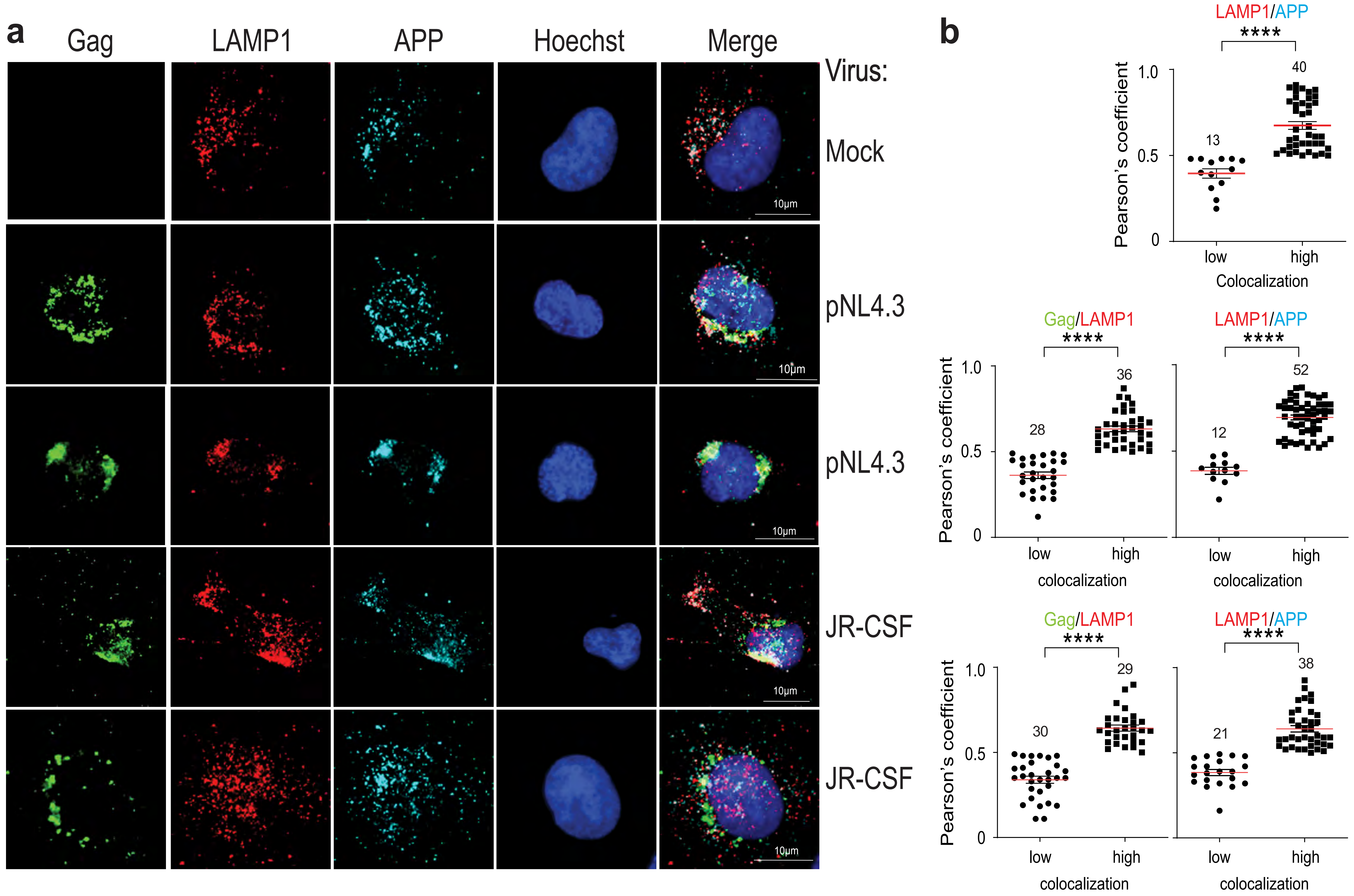

Supplementary Fig. 2. HIV-1 redistributes LAMP1-positive APP-containing vesicles. a. Representative images of APP and LAMP1 staining in CHME3 4X4 infected with mock, or either pNL4.3- or JR-CSF-derived HIV-1 at 16 h.p.i **b**. Gag or APP overlap with LAMP1 was determined by Pearson's Correlation Coefficient; mean with SEM using two-tailed Mann Whitney test (Mock LAMP1/ APP graph) or unpaired two-tailed *t* test for the remaining graphs, ****p < 0.0001. Number of cells analyzed is indicated. Source data are provided as a Source Data file.

Supplementary Fig. 3

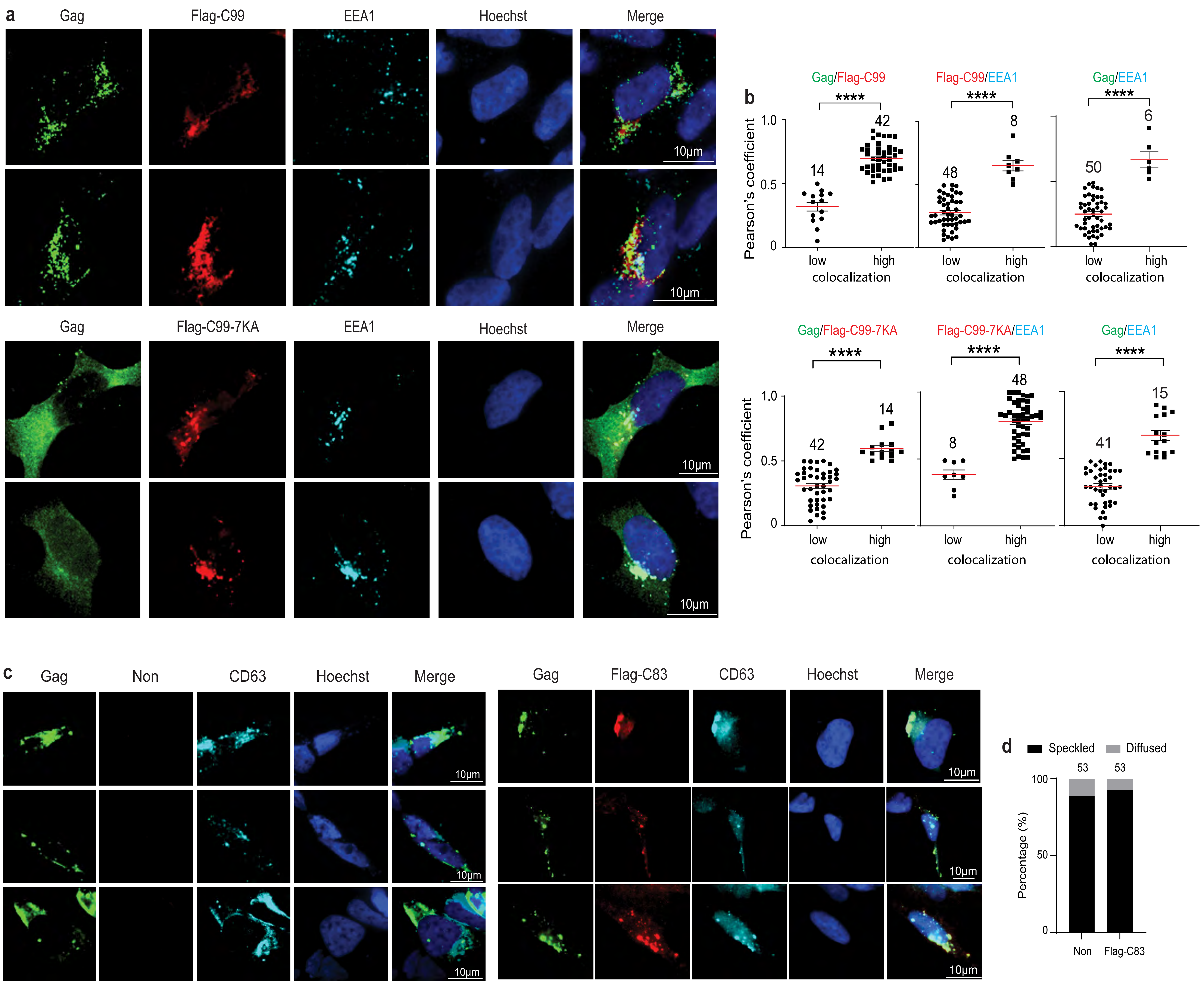

Supplementary Fig. 3. Vesicular localization patterns of Gag and CTFs in JR-CSF transfected cells. a-b. The C99-7KA mutant localizes to EEA1-positive early endosomes more strongly than C99. JR-CSF-transfected HEK293A cells transiently expressing Flag-C99 or Flag-C99-7KA mutant (upper and lower panels, respectively) were fixed and stained for Gag, Flag and EEA1, detecting nuclei using Hoechst. **a**. Representative images of cells expressing Flag-C99 or Flag-C99-7KA at 24h post-transfection. **b.** Quantification of the colocalization of Gag and C99, C99 and EEA1, or Gag and EEA1 under each condition determined by Pearson's Correlation Coefficient; mean with SEM using unpaired two-tailed *t* test, ****$p < 0.0001$. Number of cells analyzed is indicated. **c-d. C83 does not affect HIV-1 Gag localization to CD63-positive MVBs.** JR-CSF-transfected HEK293A cells transiently expressing Flag-C83 were fixed and stained for Gag, Flag and CD63, detecting nuclei using Hoechst. **c.** Representative images (n=3) of cells expressing Flag-C83 at 48h post-transfection. Examples of cells with strong C83 localization to CD63-positive vesicles are shown, highlighting its inability to affect Gag localization to these MVBs subsets. **d.** Quantification of cells exhibiting typical punctate/speckled versus diffuse Gag distribution patterns. Number of cells analyzed is indicated. Source data are provided as a Source Data file.

Supplementary Fig. 4

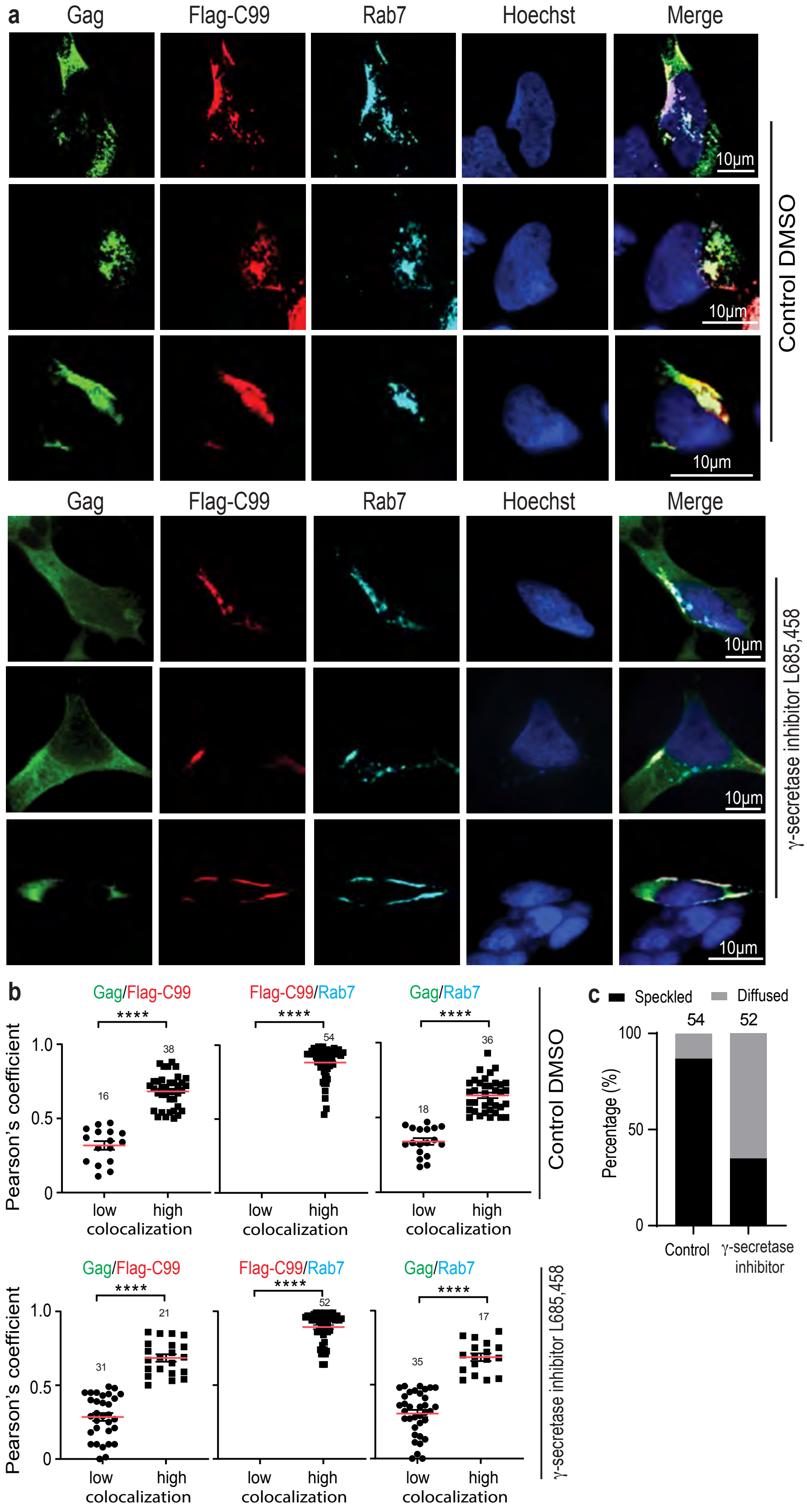

Supplementary Fig. 4. Preventing exogenous APP processing limits HIV-1 Gag entry into MVBs. HEK293A cells transfected with Flag-C99 and JR-CSF were treated with DMSO control or γ-secretase inhibitor L685,458 at 4h post-transfection. 24h post-transfection, cells were fixed and stained for Gag, Rab7 and Flag, detecting nuclei using Hoechst. **a.** Representative images of cells expressing Flag-C99 and Gag in the presence of DMSO control or L685,458. **b.** Quantification of the colocalization of Gag and C99, C99 and Rab7, or Gag and Rab7 under each condition determined by Pearson's Correlation Coefficient; mean with SEM using one sample Wilcoxon test with hypothetical value 0.50 (control and γ-secretase inhibitor Flag-C99/Rab7 graphs) or unpaired two-tailed *t* test for the remaining graphs, ****$p < 0.0001$. **c.** Quantification of cells exhibiting typical punctate/speckled versus diffuse Gag distribution patterns. Number of cells analyzed in **b** and **c** are indicated. Source data are provided as a Source Data file.

Supplementary Fig. 5

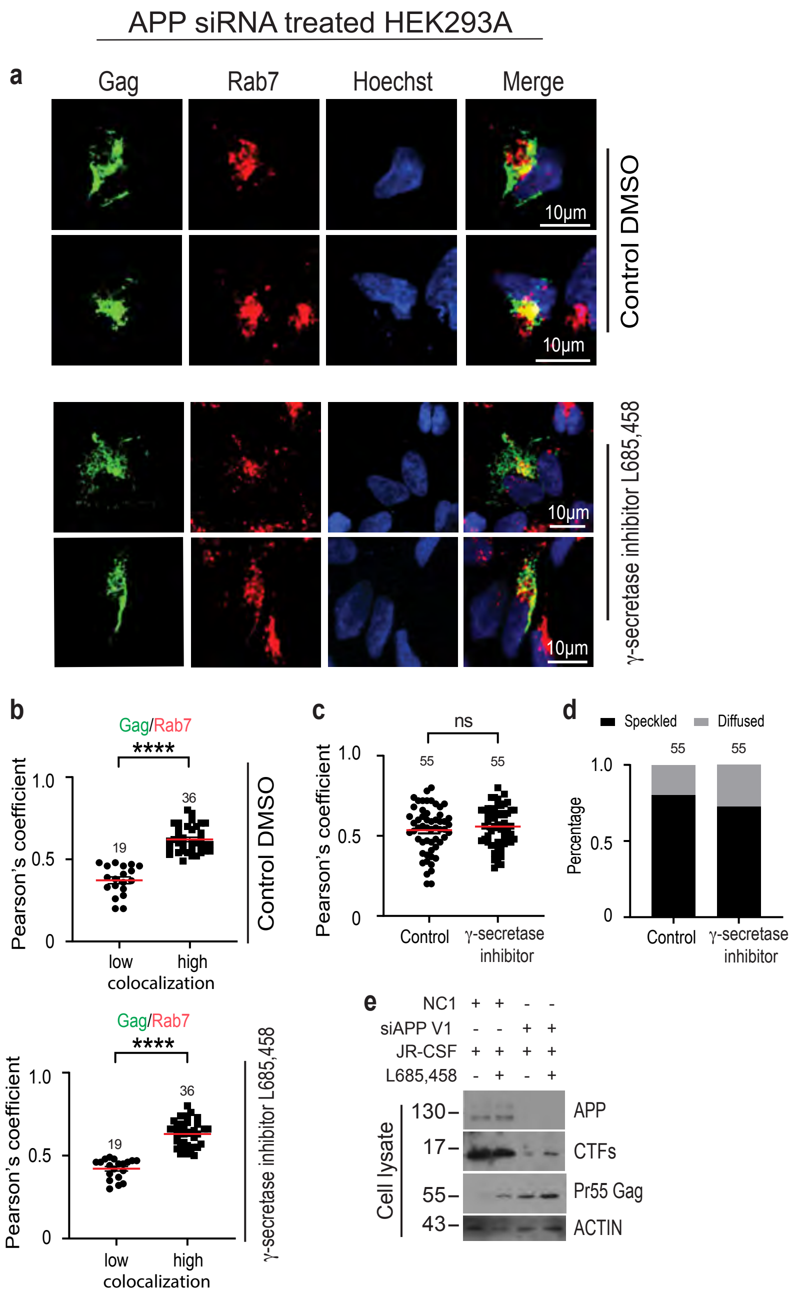

Supplementary Fig. 5. The inhibitory effect of APP processing on HIV-1 Gag sorting into Rab7-positive MVBs is reversed in cells depleted of APP. HEK293A cells treated with APP siRNAs were transfected with JR-CSF followed by treatment with DMSO control or γ-secretase inhibitor L685,458 4h post-transfection. 24h post-transfection, cells were fixed and stained for Gag, Rab7, detecting nuclei using Hoechst. **a.** Representative images of cells treated with DMSO control (upper panels) or L685,458 (lower panels). **b-c.** Quantification of the colocalization of Gag and Rab7 under each condition determined by Pearson's Correlation Coefficient; mean with SEM using unpaired two-tailed *t* test, ****$p < 0.0001$. Number of cells analyzed is indicated. Panel **c** shows the combined high and low data analysis from panel **b**. **d.** Quantification of cells exhibiting typical punctate/speckled versus diffuse Gag distribution patterns. Number of cells analyzed is indicated. **e.** Representative (n=3) WB confirmation of APP, CTFs and Pr55 Gag levels in samples from **a**. Source data are provided as a Source Data file.

Supplemental Tables

Supplementary table 1. Primer sequence information. The restriction enzyme sites are shown in bold and the tag (HA, Flag or V5 as indicated in the relevant primer name) peptide sequence is underlined.

Plasmid Name	Primer Sequence (5' to 3')
pQCXIN-GAPDH-HA	Forward: 5'-ccaact**gcggccgc**atggggaaggtgaaggtcgg-3'
	Reverse: 5'-ttgcc**gaattc**ttaagcgtaatctggaacatcgtatgggtactccttggaggccatgtgg-3'
pQCXIN-GAG-HA	Forward: 5'-ccaact**gcggccgc**accatgggtgcgagagcgtcagta-3'
	Reverse: 5'-ttgcc**gaattc**ttaagcgtaatctggaacatcgtatgggtattgtgacgaggggtcgttg-3'
pQCXIN-NCT-HA	Forward: 5'-ccaact**gcggccgc**atggctacggcagggggt-3'
	Reverse: 5'-gcaacg**ttaattaa**ttaagcgtaatctggaacatcgtatgggtagtatgacacagctcctggct-3'
pQCXIP-Flag-C99	Forward: 5'-gcaact**gcggccgc**catggactacaaagacgatgacgacaaggatgcagaattccgacatgac-3'
	Reverse: 5'-gcaacg**ttaattaa**ctagttctgcatctgctcaaag-3'
pQCXIP-Flag-C83	Forward:5'-gcaact**gcggccgc**catggactacaaagacgatgacgacaagttggtgttctttgcagaagat-3'
	Reverse: 5'-gcaacg**ttaattaa**ctagttctgcatctgctcaaag-3'
pQCXIP-C83-V5	Forward: 5'-gcaact**gcggccgc**catgttggtgttctttgcagaagat-3'
	Reverse: 5'-gcaacg**ttaattaa**ctacgtagaatcgagaccgaggagagggttagggataggcttaccgttctgcatctgctcaaag-3'
K687A	Forward: 5'-cacatcttctgcaaagaacaccaatgcttgatgatgaacttcatatcctgag-3'
	Reverse: 5'-ctcaggatatgaagttcatcatcaagcattggtgttctttgcagaagatgtg-3'
K699A	Forward: 5'-gagtccaatgattgcacctgcgtttgaacccacatcttctg-3'
	Reverse: 5'-cagaagatgtgggttcaaacgcaggtgcaatcattggactc-3'
K724,725,726A	Forward: 5'-cacaccatgatgaatggatgtgtactgtgccgccgccagcatcaccaaggtgatgacgatcact-3'
	Reverse: 5'-agtgatcgtcatcaccttggtgatgctggcggcggcacagtacacatccattcatcatggtgtg-3'
K751A	Forward: 5'-ccgttctgctgcatcgcggacaggtggcgctc-3'
	Reverse: 5'-gagcgccacctgtccgcgatgcagcagaacgg-3'
K763A	Forward: 5'-tctgcatctgctcaaagaacgcgtaggttggattttcgtagc-3'
	Reverse: 5'-gctacgaaaatccaacctacgcgttctttgagcagatgcaga-3'
pCDH-Flag-C99<br>pCDH-Flag-C99-7KA	Forward: 5'-gcaacc**tctaga**atggactacaaagacgatgacgacaaggatgcagaattccgacatgac-3'
	Reverse: 5'-gcaact**gcggccgc**ctagttctgcatctgctcaaag-3'

Supplementary table 2. siRNAs concentration and commercial information.

Cell	siRNA	Ambion ID	Concentration
HEK293A/CHME3/ MDM	Negative control 1 (NC1)	AM4635	10pmol/µL
HEK293A/CHME3	APP V1	S1500	10pmol/µL
MDM	APP V2	S1501	10pmol/µL
HEK293A	Negative control 2 (NC2)	AM4637	100pmol/µL
HEK293A	UBE1	13079	100pmol/µL
