# Supplementary material for: HIV-1 promotes ubiquitination of the amyloidogenic C-terminal fragment of APP to support viral replication
Source: Nat Commun. 2023 Jul 15;14:4227. doi: 10.1038/s41467-023-40000-x (PMC10349857; doi:10.1038/s41467-023-40000-x)
Supplement: Supplementary file 3 — Reporting Summary [file 41467_2023_40000_MOESM3_ESM.pdf]

nature portfolio

Corresponding author(s): Mojgan H. Naghavi

Last updated by author(s): Jun 23, 2023

Reporting Summary

Nature Portfolio wishes to improve the reproducibility of the work that we publish. This form provides structure for consistency and transparency in reporting. For further information on Nature Portfolio policies, see our Editorial Policies and the Editorial Policy Checklist.

Statistics

For all statistical analyses, confirm that the following items are present in the figure legend, table legend, main text, or Methods section.

n/a	Confirmed	
☐	☒	The exact sample size (*n*) for each experimental group/condition, given as a discrete number and unit of measurement
☐	☒	A statement on whether measurements were taken from distinct samples or whether the same sample was measured repeatedly
☐	☒	The statistical test(s) used AND whether they are one- or two-sided<br>*Only common tests should be described solely by name; describe more complex techniques in the Methods section.*
☒	☐	A description of all covariates tested
☒	☐	A description of any assumptions or corrections, such as tests of normality and adjustment for multiple comparisons
☐	☒	A full description of the statistical parameters including central tendency (e.g. means) or other basic estimates (e.g. regression coefficient) AND variation (e.g. standard deviation) or associated estimates of uncertainty (e.g. confidence intervals)
☐	☒	For null hypothesis testing, the test statistic (e.g. *F*, *t*, *r*) with confidence intervals, effect sizes, degrees of freedom and *P* value noted<br>*Give P values as exact values whenever suitable.*
☒	☐	For Bayesian analysis, information on the choice of priors and Markov chain Monte Carlo settings
☒	☐	For hierarchical and complex designs, identification of the appropriate level for tests and full reporting of outcomes
☐	☒	Estimates of effect sizes (e.g. Cohen's *d*, Pearson's *r*), indicating how they were calculated

Our web collection on statistics for biologists contains articles on many of the points above.

Software and code

Policy information about availability of computer code

Data collection	Microscopy imaging data were collected with Metamorph imaging software version 7.10.5.476 using Leica DMI 6000B microscope
Data analysis	Image J (version 2.3.0/1.53q) was used to quantify western blots, and the plugin Colocalization Finder was used to analyze images. GraphPad Prism 9.4.1 was used for creating graphs and doing statistical analysis. Adobe Photoshop 2022Photoshop 2022 version 23.3.0.394 and illustrator 2022 version 26.2.1 were used to create figures.

For manuscripts utilizing custom algorithms or software that are central to the research but not yet described in published literature, software must be made available to editors and reviewers. We strongly encourage code deposition in a community repository (e.g. GitHub). See the Nature Portfolio guidelines for submitting code & software for further information.

Data

Policy information about availability of data

All manuscripts must include a data availability statement. This statement should provide the following information, where applicable:

- Accession codes, unique identifiers, or web links for publicly available datasets
- A description of any restrictions on data availability
- For clinical datasets or third party data, please ensure that the statement adheres to our policy

The manuscript includes a data availability statement that the data supporting the findings of this study are available within the article and its Supplementary Information as well as in the Source Data file including graph raw data and uncropped blots.

Research involving human participants, their data, or biological material

Policy information about studies with human participants or human data. See also policy information about sex, gender (identity/presentation), and sexual orientation and race, ethnicity and racism.

Reporting on sex and gender	n.a
Reporting on race, ethnicity, or other socially relevant groupings	n.a
Population characteristics	n.a
Recruitment	n.a
Ethics oversight	n.a

Note that full information on the approval of the study protocol must also be provided in the manuscript.

Field-specific reporting

Please select the one below that is the best fit for your research. If you are not sure, read the appropriate sections before making your selection.

☒ Life sciences ☐ Behavioural & social sciences ☐ Ecological, evolutionary & environmental sciences

For a reference copy of the document with all sections, see nature.com/documents/nr-reporting-summary-flat.pdf

Life sciences study design

All studies must disclose on these points even when the disclosure is negative.

Sample size	No sample size calculation was performed. Sample size was determined based on standards for cell biology. Cell samples were collected from at least 3 biological replicates to detect protein expression by western blot with sufficient reproducibility. For imaging purposes, approximately 50 cells were analyzed for each sample, and there were at least 2 biological replicates for each imaging experiment. For each condition, 3 different cell type were tested, which were considered adequate for the experimental objectives and provide enough confidence on the conclusions.
Data exclusions	No data were excluded.
Replication	All experimental findings were replicated at least 3 times. All attempts at replication were successful.
Randomization	This was a hypothesis-driven study without intervention. No animal model or human clinical trial was involved. Cell set up was homogenized. Randomization is not relevant.
Blinding	This was a hypothesis-driven study without intervention. No subjective allocation was involved. Blinding is not relevant to this study.

Reporting for specific materials, systems and methods

We require information from authors about some types of materials, experimental systems and methods used in many studies. Here, indicate whether each material, system or method listed is relevant to your study. If you are not sure if a list item applies to your research, read the appropriate section before selecting a response.

Materials & experimental systems

n/a	Involved in the study	
☐	☒	Antibodies
☐	☒	Eukaryotic cell lines
☒	☐	Palaeontology and archaeology
☒	☐	Animals and other organisms
☒	☐	Clinical data
☒	☐	Dual use research of concern
☒	☐	Plants

Methods

n/a	Involved in the study	
☒	☐	ChIP-seq
☒	☐	Flow cytometry
☒	☐	MRI-based neuroimaging

Antibodies

Antibodies used

APP/CTFs Y188 (ab32136, Abcam, Western blotting 1:1000), APP (LN27, Invitrogen, 130200, Immunofluorescence assay 1:150), ACTIN (A2103, Sigma, Western blotting 1:1000), PARP (9542, Cell Signaling Technology, Western blotting 1:1000), V5 (D3H8Q, 13202, Cell Signaling Technology, Western blotting 1:1000), HIV-1 Pr55/p24/p17(ab63917, Abcam, Western blotting 1:1000, Immunofluorescence assay 1:200) (labeled as Pr55 Gag in the Figures), HIV-1 p24 (ab9071, Abcam, Immunofluorescence assay 1:200), Ubiquitin (P4D1, 3936, Cell Signaling Technology, Western blotting 1:1000), Flag (F7425, Sigma, Western blotting 1:1000), Flag (L5, NBP1-06712, Novus, Immunofluorescence assay 1:100), Flag (M2, Sigma, see Immunoprecipitation assay section), HA (H3663, Sigma, Western blotting 1:1000), Rab7 (D95F2, 9367, Cell Signaling Technology, Immunofluorescence assay 1:200), EEA1 (C45B10, 3288, Cell Signaling Technology, Immunofluorescence assay 1:200), CD63 (H5C6, DSHB, Immunofluorescence assay 1:100), LAMP1 (1D4B, sc-19992, Santa Cruz, Immunofluorescence assay 1:100) and UBE1 (15912-1-AP, Proteintech, Western blotting 1:1000) were used according to manufacturer's instructions. Secondary HRP linked antibodies against Rabbit IgG (NA934) and Mouse IgG (NA931) were obtained from GE Healthcare UK and used at 1:10000 dilution. Alexa fluorescence-conjugated secondary antibodies against Mouse IgG 647 (Invitrogen, A31571), Mouse IgG 488 (Invitrogen, A21202), Mouse IgG 555 (Invitrogen, A31570), Rabbit IgG 647 (Invitrogen, A31573), Rabbit IgG 488 (Invitrogen, A21206), Rabbit IgG 555 (Invitrogen, A31572), Rat IgG 647 (Jackson ImmunoResearch, 712-605-150) were used at 1:400 dilution.

Validation

1. Y188 (ab32136, Abcam): Produced recombinantly (animal-free) for high batch-to-batch consistency and long term security of supply; Rabbit monoclonal [Y188] to Amyloid Precursor Protein ;Suitable for: WB, IHC-P, IP, ICC/IF; Knockout validated; Reacts with: Mouse, Rat, Human.

2. APP (LN27, Invitrogen, 130200): Species Reactivity:Human; this Antibody was verified by Relative expression to ensure that the antibody binds to the antigen stated. Immunofluorescence assay was tested in Chai Q, Nat Commun. 2017 doi: 10.1038s41467-017-01795-8.

3. ACTIN (A2103, Sigma): Anti-Actin, N-terminal antibody produced in rabbit has been used in immunoblotting; species reactivity. frog, rat, mouse, chicke:n, human.

4. PARP (9542, Cell Signaling Technology): PARP Antibody detects endogenous levels of full length PARP1 (116 kDa), as well as the large fragment (89 kDa) of PARP1 resulting from caspase cleavage. The antibody does not cross-react with related proteins or other PARP isoforms. Species Reactivity: Human, Mouse, Rat, Monkey. Application Dilution: Western Blotting 1:1000, Simple Western™ 1:10 - 1:50.

5. V5 (D3H8Q, 13202, Cell Signaling Technology): V5-Tag (D3H8Q) Rabbit mAb recognizes transfected levels of recombinant protein containing the V5 epitope tag. Species Reactivity: All Species Expected. Application Dilution: Western Blotting 1:1000, Immunoprecipitation 1:50, Immunofluorescence (Immunocytochemistry) 1:500 - 1:2000, Flow Cytometry (Fixed/Permeabilized) 1:400 - 1:1600.

6. HIV-1 Pr55/p24/p17(ab63917, Abcam): Rabbit polyclonal to HIV1 p55 + p24 + p17. suitable for: WB, ELISA. Reacts with: Human immunodeficiency virus, Isotype: IgG.

7. HIV-1 p24 (ab9071, Abcam): Human Immunodeficiency Virus Type 1(HIV 1) p24 protein. No detectable reaction has been observed with HIV-2 or SIV (Simian Immunodeficiency Virus) viral lysates by ELISA or Western blot. Tested applications Suitable for: WB, Radioimmunoprecipitation, ELISA, ICC/IF, Sandwich ELISA. Species reactivity Reacts with: Species independent. Use a concentration of 1 - 10 µg/ml. Exhibits reactivity with HIV-1 infected cultures using indirect immunofluorescence.

8. Ubiquitin (P4D1, 3936, Cell Signaling Technology):REACTIVITY:All. SENSITIVITY Endogenous:Source/Isotype Mouse IgG1. Application Dilution: Western Blotting 1:1000, Immunohistochemistry (Paraffin) 1:100 - 1:400.

9. Flag (F7425, Sigma): The rabbit Anti-FLAG polyclonal affinity antibody ANTI-FLAG recognizes the FLAG epitope located on FLAG fusion proteins. This antibody reacts with N-terminal, N-terminal-Met, and C-terminal FLAG fusion proteins. Applying dot blot, immunoblotting, immunoprecipitation and immunocytochemistry assays.

10. Flag (L5, NBP1-06712, Novus): DYKDDDDK Epitope Tag Antibody (L5) was made to N-terminal DYKDDDDK-tagged extracellular domain of mouse Langerin. Binds to same epitope as Sigma's Anti-FLAG® M2 Antibody. Host: Rat. This DYKDDDDK Epitope Tag antibody is useful for Immunocytochemistry/Immunofluorescence, Immunoprecipitation, Western blot and Immunohistochemistry.

11. Flag (M2, Sigma): Anti Flag M2 antibody is used for the detection of Flag fusion proteins. This monoclonal antibody is produced in mouse and recognizes the FLAG sequence at the N-terminus, Met N-terminus, and C-terminus. The antibody is also able to recognize FLAG at an internal site. Monoclonal ANTI-FLAG® M2 antibody produced in mouse has been used in: immunoblotting, immunoprecipitation, immunocytochemistry,immunofluorescence, ELISA, EIA, chromatin immunoprecipitation electron microscopy, flow cytometry, supershift assays.

12. HA (H3663, Sigma): The antibody recognizes native as well as denatured-reduced forms of HA-tagged proteins and is reactive with N- or C-terminal HA-tagged fusion proteins expressed in E. coli or in mammalian cells. Monoclonal Anti-HA antibody produced in mouse has been used: in immunoblotting, immunocytochemistry, immunoprecipitation.

13. Rab7 (D95F2, 9367, Cell Signaling Technology): Rab7 (D95F2) XP® Rabbit mAb detects endogenous levels of total Rab7 protein. Species Reactivity:Human, Mouse, Rat, Monkey. Application Dilution: Western Blotting 1:1000, Immunoprecipitation 1:50, Immunofluorescence (Immunocytochemistry) 1:50 - 1:200.

14.EEA1 (C45B10, 3288, Cell Signaling Technology): EEA1 (C45B10) Rabbit mAb detects endogenous levels of total EEA1 protein. Species Reactivity:Human, Mouse, Rat. Application Dilution: Western Blotting 1:1000, Immunoprecipitation 1:100, Immunofluorescence (Frozen) 1:50 - 1:200, Immunofluorescence (Immunocytochemistry) 1:50 - 1:200.

15. CD63 (H5C6, DSHB): Antigen Species: Human. The concentration for immunohistochemistry (IHC), immunofluorescence (IF), and immunocytochemistry (ICC) when using mouse Ig is 2-5 ug/ml.
16. LAMP1 (1D4B, sc-19992, Santa Cruz): LAMP-1 (1D4B) is a rat monoclonal antibody raised against NIH/3T3 mouse embryo fibroblast tissue culture cell membranes. LAMP-1 (1D4B) is recommended for detection of LAMP-1 of mouse, rat and human origin by Western Blotting (starting dilution 1:200, dilution range 1:100-1:1000), immunoprecipitation [1-2 µg per 100-500 µg of total protein (1 ml of cell lysate)], immunofluorescence (starting dilution 1:50, dilution range 1:50-1:500).
17. UBE1 (15912-1-AP, Proteintech): tested Reactivity: Human, Mouse, Rat. Application: Western Blot :1:500-1:3000, Immunohistochemistry : 1:50-1:500. Knockdown validated.

Eukaryotic cell lines

Policy information about cell lines and Sex and Gender in Research

Cell line source(s)

HEK293A were generated and validated by Jeremy Luban lab (University of Massachusetts Medical School, Massachusetts)
HEK293T cells were obtained from ATCC (cat #CLR-3216)
HeLa-TZM-bl cells were obtained from NIH AIDS Reagent Program (no. 8129)
CHME3 was obtained from Dr. Marc Tardieu lab (Universite Paris Sud, France) where they were originated and validated
CHME3 4X4 were generated and validated by Olivier Schwarts lab (Pasteur Institute, France)
Peripheral blood mononuclear cells (PBMCs) were obtained from New York blood center

Authentication

All cell lines were authenticated.

Mycoplasma contamination

All cell lines were tested negative for Mycoplasma.

Commonly misidentified lines
(See ICLAC register)

No misidentified cell lines were used in this study.
